# SOCIAL ISOLATION AND DEMENTIA ONSET: THE ROLE OF DENTAL VISITS AND TOOTH STATUS

**DOI:** 10.1093/geroni/igac059.1099

**Published:** 2022-12-20

**Authors:** Xiang Qi, Bei Wu

**Affiliations:** New York University, New York City, New York, United States; New York University, New York, New York, United States

## Abstract

Using data from the Health and Retirement Study, this study investigated the mediating effects of dental visits and tooth status (measured by edentulism) on the association between social isolation and dementia onset. Social isolation (exposure) and covariates in 2008, mediators (dental visits and edentulism) in 2012, and the onset of dementia between 2012 and 2018 were obtained. Dementia was identified through self- or proxy-reported physician diagnosis. We included 8,744 participants, and 576 (6.6%) had dementia during follow-up. There was a significant effect of social isolation on the onset of dementia (Hazard Ratio [HR], 1.14; 95% CI, 1.01-1.28). Controlling for mediators, the effect of social isolation was reduced to 1.10 (95% CI, 0.98-1.25), leaving an indirect effect of 1.03 (95% CI, 1.02-1.04). The proportions mediated by dental visits and edentulism were 4.4% and 7.5%, respectively. Our findings highlight the importance of improving oral health and dental care for older adults.

